# Association of Surfactant-Associated Protein D Gene Polymorphisms with the Risk of COPD: a Meta-Analysis

**DOI:** 10.6061/clinics/2019/e855

**Published:** 2019-05-13

**Authors:** Yi Liao, ChengLiang Huang, JianRong Wang, XianMing Fan

**Affiliations:** Department of Respiratory Medicine, The Affiliated Hospital of Southwest Medical University, Luzhou, Sichuan 646000, P.R. China

**Keywords:** Surfactant-Associated Protein D, Chronic Obstructive Pulmonary Disease, Genetic Polymorphism, Meta-Analysis

## Abstract

The relationship between surfactant-associated protein D polymorphisms and chronic obstructive pulmonary disease risk remains controversial. This article is the first to systematically evaluate this relationship. A comprehensive worldwide search was conducted for relevant literature on surfactant-associated protein D gene mutations and chronic obstructive pulmonary disease risk prediction. Study quality was evaluated using the Newcastle-Ottawa scale. After four genetic models (the allele, additive, recessive, and dominant models) were identified, odds ratios (ORs) and the corresponding 95% confidence intervals (CIs) were applied in this meta-analysis. The meta-analysis included 659 individuals in the case group and 597 in the control group. In the Asian population, none of the four genetic models revealed any significant association between rs2243639 genotype and the risk of chronic obstructive pulmonary disease. In Caucasians, however, the recessive model exhibited significant risk associated with rs2243639. Furthermore, there was a significant association between rs721917 genotype and the risk of chronic obstructive pulmonary disease in the Asian population. In contrast, none of the four gene models revealed any significant risk associated with this gene in the Caucasian population. This meta-analysis suggests that rs2243639 is not related to the risk of chronic obstructive pulmonary disease in the Asian population but is related to this risk in the Caucasian population. Regarding rs721917, the T allele may increase the risk of chronic obstructive pulmonary disease in the Asian population.

## INTRODUCTION

Chronic obstructive pulmonary disease (COPD) is a preventable, treatable disease characterized by persistent airflow limitation accompanied by gradual deterioration of lung function and a series of abnormalities in mental and physical functions 1. COPD imposes a heavy economic burden on global public health resources. It is one of the major causes of hospitalization and emergency care, and it is also one of the leading causes of death worldwide 2,3. Approximately 3.2 million people died in 2015 due to COPD and related complications 4.

The pathogenesis of COPD is still not fully understood. It is generally believed to be related to inflammation, protease-antitrypsin imbalance, and oxidative stress stimulation. It is widely believed that COPD is caused by both environmental and genetic factors. In recent years, with the development of genome-wide association study (GWAS) technology, increasing numbers of COPD-related susceptibility genes have been discovered, such as tumor necrosis factor-α (TNF- α), transforming growth factor-β1 (TGF-β1), A disintegrin and metalloproteinase 33 (ADAM33), superoxide dismutase (SOD), alveolar surface-associated protein D (SP-D) and other related susceptibility genes 5.

Alveolar surfactant-associated protein (SP) is an active substance synthesized and secreted by alveolar type II epithelial cells. It is composed of phospholipids and a small amount of protein, and it usually covers the alveolar surface to maintain alveolar surface tension and promote the smooth progress of pulmonary ventilation. It also has immunomodulatory and anti-inflammatory effects 6. SP has four phenotypes, namely, A, B, C and D. Among them, D plays a prominent role in host immune regulation. A study confirmed that the expression level of SP-D in the serum can be used as a biomarker for the diagnosis of COPD 7.

Increasing studies have been performed to assess polymorphisms of the SP-D gene, but some controversies remain. The data on SP-D single nucleotide polymorphisms (SNPs) has primarily focused on rs2243639 and rs721917. Therefore, this meta-analysis was performed to explore the relationship between the abovementioned SNPs and the risk of developing COPD.

## MATERIALS AND METHODS

### Study identification

#### Scaling strategies

**Figure 1:**
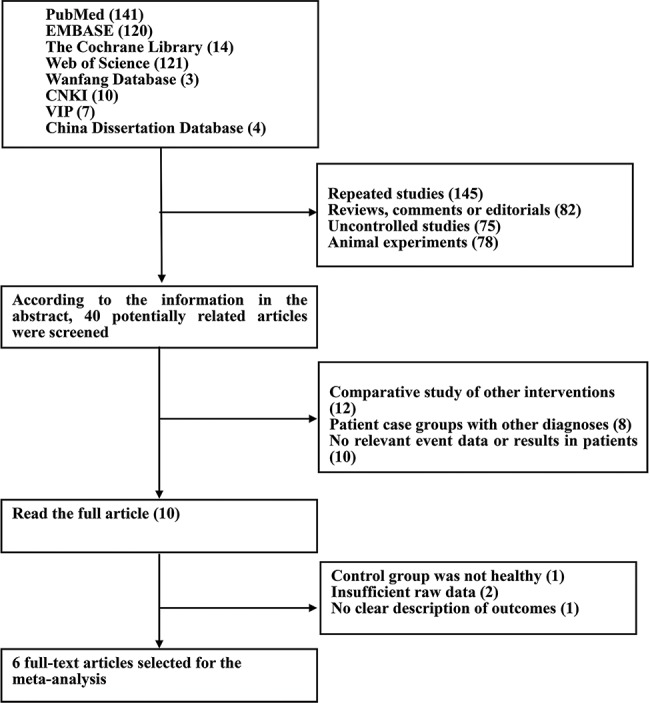
Flow chart of study selection based on the inclusion criteria.

A comprehensive search was conducted for relevant literature published by scholars worldwide on SP-D gene mutations and COPD risk prediction. The databases searched were the Wanfang Data Knowledge Service Platform and the VIP Chinese Science and Technology Periodical Database (VIP), the China National Knowledge Infrastructure (CNKI), the China Dissertation Database, PubMed, Embase, the Cochrane Library, and Web of Science. The retrieval time was set from January 1, 1997, to September 30, 2018. The search terms were “surfactant-related protein D gene” and “chronic obstructive pulmonary disease”. The results were limited to articles in English and Chinese. The literature screening process is shown in Figure 1.

The inclusion criteria were as follows: 1) The article was related to polymorphisms of the SP-D gene and the risk of COPD, 2) the study was a case-control, cohort, or group design study, 3) the inclusion and exclusion criteria of the case and control groups in the study were clear, and the experimental goals included were in line with the aim of this meta-analysis, 4) there were complete experimental data and results, 5) The case group and the control group involved human subjects, and 6) the article was written in English or Chinese.

The exclusion criteria were as follows: 1) duplicate report, 2) defects and poor-quality study design, 3) incomplete data and unclear outcome effect, 4) flawed statistics and the inability to extract relevant data, 5) animal experiments, and 6) non-Chinese- and English-language articles.

### Data extraction

The two researchers independently screened the literature to exclude the literature that did not meet the inclusion criteria. Differences were resolved through group discussion. Data were extracted independently according to the predesigned form, and the extracted data were cross-checked. The following information was extracted from each study: name of the first author, year of publication, race, definition and source of the control and case groups, smoking status in cases and controls, and the number of genotypes and individual base pairs.

### Quality assessment

The quality of the included literature was evaluated by two independent researchers in strict accordance with the Newcastle-Ottawa Scale (NOS) 8. The selectivity, comparability and exposure of each article were assessed, and all six documents included were of high quality. The literature quality assessment is shown in Table 1.

### Statistical methods

All data included in the literature were analyzed using Stata MP13.0 (64-bit). At the same time, to reduce the probability of type I errors 9, we performed multiple pairwise comparisons of each genotype and used the following four models: 1. The allele model (A *vs* B), 2. The additive model (AA *vs* BB), 3. The recessive model (AA *vs* AB+BB), and 4. The dominant model (AA+AB *vs* BB) 10. At the same time, a test was performed on the included data to further reduce sampling bias by determining if the data were in Hardy-Weinberg equilibrium (HWE) 11. In addition, the minor allele frequency (MAF) was calculated. Heterogeneity tests were performed on all included data. If the heterogeneity was not significant (*p*>0.05, *I^2^*<50%), the M-H fixed effect model was used to calculate the odds ratio (OR) and its 95% confidence interval (CI). If the heterogeneity was significant (*p*<0.05, *I^2^*>50%), the OR and its 95% CI were randomly calculated using the M-H heterogeneity random effects model. At the same time, subgroup analyses were performed using different ethnic classifications. All included articles were analyzed for bias using the Egger method. If *p*<0.05, publication bias was considered. The risk of bias is shown in Table 2. The gene distribution of each model is shown in Table 3.

## RESULTS

### Study characteristics

In total, 420 articles were initially collected, and 380 articles that did not meet the inclusion criteria were then excluded. Thirty articles that had defects in experimental design, case-control criteria, and other aspects were excluded based on the information in the abstract. After reading the full texts of the remaining articles, 4 additional articles were excluded. In total, 6 articles 12-17 were included, with 2 written in Chinese and 4 in English. The meta-analysis included 9 groups in case-control studies, including 659 individuals in the case group and 597 in the control group, for a total of 1256 individuals. Two sets of case-control studies were from Europe, and the rest were from Asia. The two SNPs investigated were rs2243639 and rs721917. The characteristics of the included studies are shown in Table 4.

### Association between SPD gene polymorphisms and COPD

**Figure 2:**
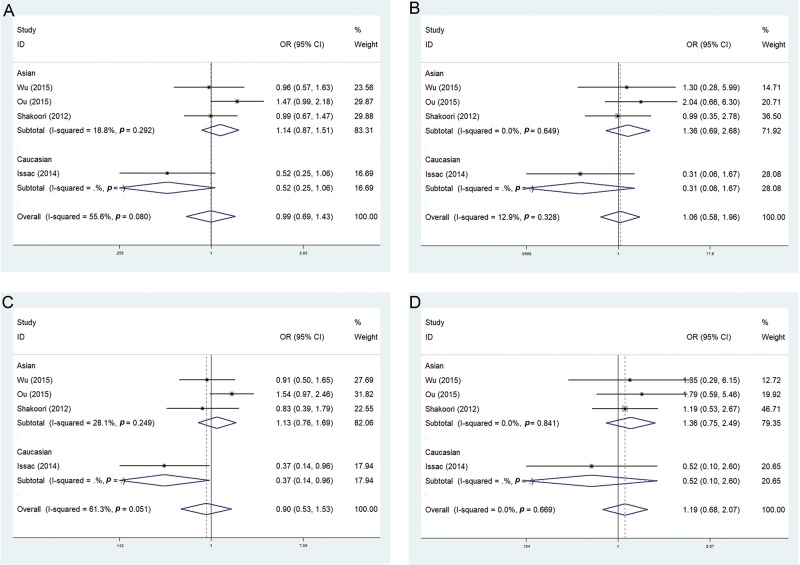
Forest plots of the association between the rs2243639 polymorphism and the risk of COPD (A, allelic model; B, additive model; C, recessive model; D, dominant model).

The total population analysis of rs2243639 with the random effects model revealed heterogeneity in the allelic model (OR 0.99; 95% CI 0.69-1.43; I^2^=55.6%) and recessive model (OR 0.90; 95% CI 0.53- 1.53; I^2^=61.3%). When using the fixed effect model, there was low heterogeneity in the additive model (OR 1.06; 95% CI 0.58-1.96; I^2^= 12.9%) and the dominant model (OR 1.19; 95% CI 0.68-2.07; I^2^=0%). In the subgroup analysis in the Asian population, the allelic model (OR 1.14; 95% CI 0.87-1.15; I^2^=18.8%), additive model (OR 1.36; 95% CI 0.69-2.68; I^2^=18.8%), recessive model (OR 1.13; 95% CI 0.76-1.69; I^2^=28.1%), and dominant model (OR 1.36; 95% CI 0.75-2.49; I^2^=0%) revealed no association. However, for Caucasians, the recessive model was significantly different (OR 0.37; 95% CI 0.14-0.96; I^2^=0%) (Figure 2) (Table 5).

**Figure 3:**
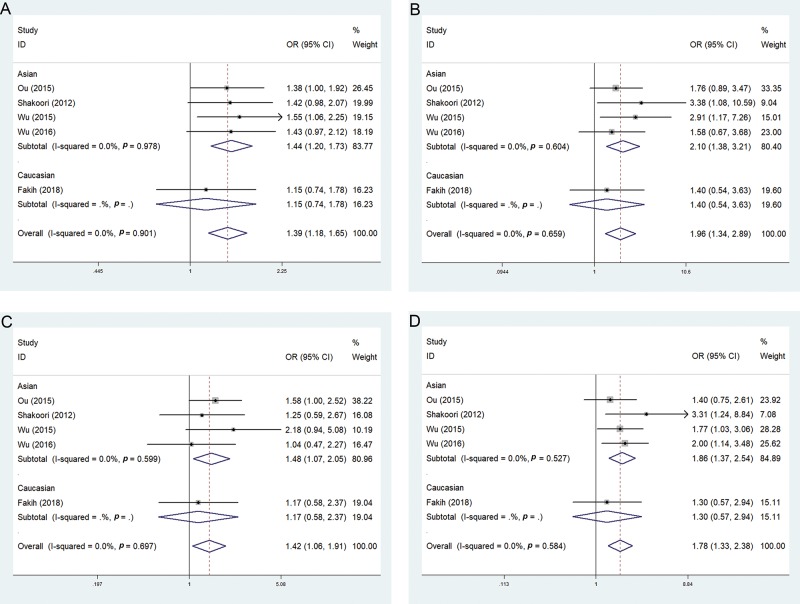
Forest plots of the association between the rs721917 polymorphism and the risk of COPD (A, allelic model; B, additive model; C, recessive model; D, dominant model).

For the total population analysis of rs721917, when using a fixed effect model, the allelic model (OR 1.39; 95% CI 1.18-1.65; I^2^=0%), additive model (OR 1.96; 95% CI 1.34-2.89; I^2^=0%), recessive model (OR 1.42; 95% CI 1.06-1.91; I^2^=0%), and dominant model (OR 1.78; 95% CI 1.33-2.38; I^2^=0%) had low heterogeneity. In the subgroup analysis, in the Asian population, there was low heterogeneity in the allele model (OR 1.44; 95% CI 1.20-1.73; I^2^=0%), additive model (OR 2.10; 95% CI 1.38-3.21; I^2^=0%), recessive model (OR 1.48; 95% CI 1.07-2.05; I^2^=0%), and dominant model (OR 1.86; 95% CI 1.37-2.54; I^2^=0%). There were no significant differences in the risk of COPD in the Caucasian population in the four gene models (Figure 3) (Table 5).

### Publication bias

Publication bias was assessed using Egger's test. We detected significant publication bias in the additive model for rs2243639 in the total population only. There was no significant evidence of publication bias in the other genetic models (Table 2).

## DISCUSSION

Previous studies have shown that smoking is a risk factor for COPD development but that genetic factors also contribute 18-25. Some researchers have studied the association between SPD polymorphisms and COPD susceptibility; this association was first reported in the Mexican population in 2001 26. Some studies have reported that the SPD gene plays an important role in the development of asthma 15, silicosis 27 and tuberculosis 28. However, there is still controversy concerning the genetic associations of SPD with COPD at present. Therefore, we attempted to collect the articles related to the genetic associations of SPD with COPD to conduct a comprehensive meta-analysis to assess this relationship accurately. Furthermore, in Asian populations, the results of the analysis of the four genotypes all indicated significant differences between the case group and the control group and low heterogeneity of rs721917. Therefore, we conclude that in Asian populations, there is a strong correlation between SPD and the risk of developing COPD.

This meta-analysis showed that there was no statistically significant association between the polymorphisms of rs2243639 and the risk of COPD in the Asian population. There were three genotypes (GG, GA, and AA) in the polymorphic locus of rs2243639 in the exon 5 region of the SP-D gene. For the Asian population, the rs2243639 polymorphism was found in both patients with COPD and healthy individuals. There was no significant difference in the risk of COPD between those with the polymorphism and those without. Surprisingly, subgroup analysis revealed that SPD polymorphisms, particularly the G allele, increased the risk of susceptibility to COPD in Caucasians. Nevertheless, only one genetic model was significantly different, the sample size of the Caucasian population was small, and only one group was included in the meta-analysis. Therefore, caution should be taken in the interpretation of the research results, and a larger sample size and well-designed investigation will be necessary to confirm the conclusion of this study.

For rs721917, the rs721917 polymorphism and the risk of COPD were significantly associated in the Asian population but not the Caucasian population. In the SP-D exon 1 region, there are three rs721917 genotypes (TT, TC, CC). The included studies and this meta-analysis showed that compared to the C allele, the T allele may increase the risk of COPD in the Asian population but not in the Caucasian population. Foreman et al. 29 demonstrated that the rs721917 polymorphism was not associated with the risk of COPD in a controlled trial of 823 patients with COPD and 810 healthy smokers in a Norwegian population. Interestingly, regarding the Asian population, Ishii et al. 30 also verified that the C allele of rs721917 is a risk genotype for COPD in the Japanese population. The reason for these reports may be as follows. 1 Due to differences in genetic background and gene distribution proceeding from the influences of region, race and environment, the results may be inconsistent. 2 Multigene interactions may also contribute; COPD results from the joint action of multiple genes and the environment. Other COPD-related genes may affect the experimental results. 3 Clinical phenotype inconsistency may contribute; although the sample size of Foreman et al. 29 was large enough to achieve high statistical power, the clinical phenotype in focus involved patients with bilateral lung emphysema, and the estimated FEV1 value was less than 45%, whereas the other reports included COPD patients whose estimated FEV1 values were greater than 45%. 4 Different groups and inclusion criteria among different studies may lead to different results.

This meta-analysis had some deficiencies. First, the number of included studies was relatively small, and the included studies were all written in Chinese or English. Useful studies in other languages may have been missed. At the same time, papers from academic conferences and other documents were not included. Second, the analysis of publication bias revealed bias in the recessive model of the rs2243639 polymorphism, and the HWE test showed that some of the included samples did not adhere to the Hardy–Weinberg principle. Third, data from Caucasian populations were scarce. At the same time, the proportion of Chinese individuals among those from Asian populations was relatively high, which had an impact on the conclusions. Fourth, in some of the included studies, there were statistically significant differences in smoking status between the case group and the control group, which also affected the results.

According to the results of the current meta-analysis, the rs2243639 polymorphism was associated with the risk of COPD in Caucasian populations but not in Asian populations. Among Asians, compared with the C allele for rs721917, the T allele may increase the risk of COPD. Due to the limitations of the quality and quantity of the articles included in the present meta-analysis, the conclusions obtained need to be confirmed by more uniform case–control or cohort studies.

## ACKNOWLEDGMENTS

We express our thanks for the English editing services of American Journal Experts.

## Figures and Tables

**Table 1:** Quality assessment of the studies included in the meta-analysis.

First author	Selection				Comparability		Exposure			Total score
	1	2	3	4	1	2	1	2	3	
Ou et al. [12]	☆	☆	-	☆	☆	☆	☆	☆	☆	8
Shakoori et al. [13]	☆	☆	☆	☆	☆	-	☆	☆	☆	7
Issac et al. [14]	☆	☆	-	☆	☆	☆	☆	☆	☆	8
Fakih et al. [15]	☆	☆	☆	☆	☆	☆	☆	☆	☆	8
Wu [16]	☆	☆	-	☆	☆	☆	☆	☆	☆	8
Wu [17]	☆	☆	-	☆	☆	-	☆	☆	☆	7

Selection: 1. Whether the case is sufficiently defined (if some independent validation was required, one star); 2. Whether the case is representative (if yes, one star); 3. The choice of the control (if they were community controls, one star); 4. Definition of control (if they had no history and new occurrence, one star). Comparability: 1. The study controlled for the influence of age (if yes, one star); 2. The study also controlled other important confounding factors such as gender and smoking history (if yes, one star). Exposure: 1. Determination of exposure (if by reliable method, one star); 2. Same method of ascertainment for cases and controls (if yes, one star); 3. No response rate (if the two groups had the same nonresponse rate, one star).

**Table 2:** Egger’s linear regression test to measure funnel plot asymmetry.

SNP	Group	P			
		A *vs* B	AA *vs* BB	AA *vs* AB+BB	AA+AB *vs* BB
rs2243639	All (n=4)	0.610	0.425	0.040	0.620
	Asian (n=3)	0.689	0.899	0.280	0.677
	Caucasian (N=1)	-	-	-	-
rs721917	All (n=5)	0.375	0.384	0.715	0.634
	Asian (n=4)	0.457	0.221	0.828	0.319
	Caucasian (N=1)	-	-	-	-

**Table 3:** Frequency of the rs2243639 and rs721917 polymorphisms in different populations.

SNP	Author	Race	Case group			Control group			HWE (*p* value)	MAF
			AA	AB	BB	AA	AB	BB		
rs2243639	Wu [16]	Asian	87	27	3	89	24	4	0.16	0.14
	Ou et al. [12]	Asian	133	53	6	76	45	7	0.92	0.19
	Shakoori et al. [13]	Asian	16	81	14	15	61	13	<0.05	0.49
	Issac et al. [14]	Caucasian	18	36	9	13	10	2	0.97	0.39
rs721917	Ou et al. [12]	Asian	86	81	25	43	62	22	0.38	0.37
	Shakoori et al. [13]	Asian	18	56	6	16	51	18	<0.05	0.47
	Fakih et al. [15]	Caucasian	17	35	10	28	64	23	0.22	0.46
	Wu [16]	Asian	18	66	33	9	60	48	0.10	0.38
	Wu [17]	Asian	15	61	34	14	42	50	0.28	0.37

HWE: Hardy-Weinberg equilibrium

MAF: Minimum allele frequency

**Table 4:** Main characteristics of the studies included in this meta-analysis.

						Age		Sex				
								Case group		Control group		
Author (year)	Race	Case group inclusion criteria	Control group inclusion criteria	Control source	Gene detection method	Case group	Control group	Male	Female	Male	Female	Smoking status (*p*-value)
Ou et al. (2015) [12]	Asian	FEV1/FVC<70% (GOLD stage 1 and higher)	Healthy	HB	PCR	68.6±11.4	58.3±12.8	192	0	128	0	*p*=0.18
Shakoori et al. (2012) [13]	Asian	FEV1/FVC<70% (GOLD stage 1 and higher)	Healthy	PB	PCR	61±13	37±11	115	0	106	0	*p*=0.11
Issac et al.(2014) [14]	Caucasian	FEV1/FVC<70% (GOLD stage 1 and higher)	Healthy	HB	PCR	56.81±9.72	54.92±7.12	63	0	25	0	*p*=0.41
Fakih et al. (2018) [15]	Caucasian	FEV1/FVC<70% (GOLD stage 1 and higher)	Healthy	PB	PCR	62 (50, 71)*	36 (23, 49)*	52	38	87	132	*p*>0.05
Wu (2015) [16]	Asian	FEV1/FVC<70% (GOLD stage 1 and higher)	Healthy	PB	PCR	66.13±5.28	66.03±7.40	104	13	97	20	*p*>0.05
Wu (2016) [17]	Asian	FEV1/FVC<70% (GOLD stage 1 and higher)	Healthy	PB	PCR	71.73±8.63	69.80±8.37	97	13	87	19	*p*<0.05

HB: hospital-based, PB: population-based, PCR: Polymerase chain reaction *All values are expressed as median (Q1;Q3); Q, quartile.

**Table 5:** Results of the meta-analysis for primary SPD SNP polymorphisms associated with the risk of COPD.

SNP	Gene model	Group	*p*	OR (95% CI)	*I^2^* (%)	Analysis model
rs2243639	A *vs* B	Asian (N=3)	0.337	1.14 (0.87-1.51)	18.8	R
		Caucasian (N=1)	0.070	0.52 (0.25-1.06)	-	R
		all (N=4)	0.976	0.99 (0.69-2.68)	55.6	R
	AA *vs* BB	Asian (N=3)	0.378	1.36 (0.69-2.69)	0	F
		Caucasian (N=1)	0.172	0.31 (0.06-1.67)	-	F
		all (N=4)	0.846	1.06 (0.58-1.96)	12.9	F
	AA *vs* AB+BB	Asian (N=3)	0.544	1.13 (0.76-1.69)	28.1	R
		Caucasian (N=1)	0.041	0.37 (0.14-0.96)	-	R
		all (N=4)	0.692	0.90 (0.53-1.53)	61.3	R
	AA+AB *vs* BB	Asian (N=3)	0.312	1.36 (0.75-2.49)	0	F
		Caucasian (N=1)	0.428	0.52 (0.10-2.60)	-	F
		all (N=4)	0.539	1.19 (0.68-2.07)	0	F
rs721917	A *vs* B	Asian (N=4)	<0.001	1.44 (1.20-1.73)	0	F
		Caucasian (N=1)	0.532	1.15 (0.74-1.78)	-	F
		all (N=5)	<0.001	1.39 (1.18-1.65)	0	F
	AA *vs* BB	Asian (N=4)	0.001	2.10 (1.38-3.21)	0	F
		Caucasian (N=1)	0.494	1.40 (0.54-3.63)	-	F
		all (N=5)	0.001	1.96 (1.34-2.89)	0	F
	AA *vs* AB+BB	Asian (N=4)	0.017	1.48 (1.07-2.05)	0	F
		Caucasian (N=1)	0.655	1.17 (0.58-2.37)	-	F
		all (N=5)	0.018	1.42 (1.06-1.91)	0	F
	AA+AB *vs* BB	Asian (N=4)	<0.001	1.86 (1.37-2.54)	0	F
		Caucasian (N=1)	0.529	1.30 (0.57-2.94)	-	F
		all (N=5)	<0.001	1.78 (1.33-2.38)	0	F

OR, odds ratio; CI, confidence interval; R, random effects model; F, fixed effects model.
